# Effect of Feeding Frequency on the Growth, Body Composition, and Intestinal Health of Hybrid Grouper (*Epinephelus fuscoguttatus*♀ × *E. lanceolatu*♂) Fed a High-Fat Diet

**DOI:** 10.3390/ani15030346

**Published:** 2025-01-25

**Authors:** Weibin Huang, Shipei Yang, Wenshan Cai, Wanting Huang, Yansheng Liu, Shuaipeng Li, Menglong Zhou, Beiping Tan, Xiaohui Dong

**Affiliations:** 1 Laboratory of Aquatic Nutrition and Feed, College of Fisheries, Guangdong Ocean University, Zhanjiang 524088, China; huangweibin311@stu.gdou.edu.cn (W.H.);; 2 Aquatic Animals Precision Nutrition and High Efficiency Feed Engineering Technology Research Center of Guangdong Province, Zhanjiang 524088, China

**Keywords:** growth performance, intestinal health, intestinal morphology

## Abstract

This experiment was to investigate the effects of feeding frequency on the growth performance, body composition, and intestinal health of hybrid grouper (*Epinephelus fuscoguttatus*♀ × *E. lanceolatu* ♂). Fifty-six days of feeding with four different feeding frequencies (1 time/day, 2 times/day, 3 times/day, and 4 times/day) were conducted on groupers with an initial body weight of 11.51 ± 0.02 g. The results show the following: (1) Weight gain rate (WGR) and specific growth rate (SGR) of the groupers in the 1 time/day group were significantly lower than in other groups (*p* < 0.05). (2) Superoxide dismutase (SOD) had the lowest value in the 1 time/day group, significantly lower than the 2 times/day and 4 times/day groups, catalase showed an upward trend, and the 4 times/day group was significantly greater than the other groups (*p* < 0.05). The total antioxidant capacity (T-AOC) and glutathione peroxidase (GPX) activities in the 1 time/day group were significantly lower than in the other groups (*p* < 0.05). (3) The increase in feeding frequency led to a significant increase in the expression levels of *cat* and *il-6 (p* < 0.05). In summary, appropriate feeding frequency can promote growth and enhance the antioxidant capacity of the fish’s gut. We recommend a best feeding frequency of 2 times/day.

## Simple Summary

Fishmeal is the most important protein source in marine fish compound feeds; however, the current market prices and the imbalance between supply and demand require us to reduce the use of fishmeal. Our previous research has demonstrated that it is feasible to reduce grouper protein to 45% with high-fat feeds. However, there is no study on the feeding frequency of grouper under this formulation model, and the feeding frequency is closely related to the culture cost and fish growth and development. In this experiment, four feeding frequencies (1~4 times/day) were designed and studied for growth and gut health. Conclusion: Appropriate feeding frequency can promote growth and enhance the antioxidant capacity of the fish’s gut. We recommend a best feeding frequency of 2 times/day.

## 1. Introduction

The hybrid grouper (*♀ Epinephelus fuscoguttatus × ♂ Epinephelus lanceolatus*) is characterized by rapid growth, strong environmental adaptability, disease resistance, and rich nutritional value [1]. It is widely cultivated in Malaysia, Taiwan, and mainland China. It has become one of the most important commercial fish species in the grouper aquaculture industry and the entire aquaculture industry, occupying a wide market share and having high economic value [2]. According to statistics, the national production of grouper in 2022 reached 205,800 tons [3].

In the cultivation of grouper, the protein content in commercial feed is about 50%, which is usually composed of high fishmeal content to meet its nutritional needs [4]. Fishmeal is an excellent source of protein with a well-balanced amino acid content and low molecular weight nitrogen compounds, and it has good palatability [5]. However, the rapid development of aquaculture and the aquafeed industry has exacerbated the imbalance between the supply and demand of fishmeal due to the unsustainability of fishery resources and the rise in fishmeal prices [6]. In fish, fat plays an important role in growth, reproduction, immune function, cell membrane function, fat substance metabolism, and the regulation of fat-soluble vitamins [7]. In order to solve the imbalance between supply and demand of fish meal, the aquaculture industry is looking for ways to reduce or substitute fishmeal [8]. Currently, there are two general approaches to reduce the amount of fish meal used in feed formulations. One is to improve the energy of the feed, such as fats or carbohydrates, thereby saving protein in the feed formulation, and since fish meal is the main protein source for saltwater fish, this can also be a good saving in the use of fish meal. However, it is worth noting that although carbohydrates have a very low cost, their effect is far less than that of high-fat content feed, which is reflected in growth effect and feed factor. Our previous study investigated the effects of different fat levels on grouper and found that a 16% high-fat diet promoted grouper growth [9]. Subsequently, we investigated different protein levels at high fat levels and found that at a 16% fat level, a protein level of 45% was appropriate, based on growth performance and enzyme activity indicators such as serum liver [10].

Fish obtain nutrients and energy through food to maintain their survival, growth, and reproduction. Feeding is influenced by many endogenous factors, including digestive and absorptive capacity as well as hormonal regulation, as well as exogenous factors such as environmental factors, food type and size, feeding frequency, and feeding rate [11,12,13]. Among these factors, feeding frequency affects bait intake, growth, metabolism, and aquaculture costs [12]. Too low a feeding frequency can lead to a decrease in fish growth and survival rates, thereby increasing the incidence of size differences and cannibalism [14]. Too high a feeding frequency can reduce the feed coefficient and increase costs [15]. As a result of previous studies, we have concluded that a ratio of 16% lipid to 45% protein is suitable for grouper, but do differences in feeding frequency on this basis have an impact on the application of high-fat diets for grouper? Therefore, due to our pre-optimization of the grouper feed formulation, a study on feeding frequency under high-fat feeds is of interest to help us better understand how to use high-fat feeds efficiently. Therefore, this experiment, combined with the practical breeding of hybrid grouper, explores the impact of feeding frequency on the growth, body composition, digestive enzyme activity, and intestinal health of juvenile hybrid grouper fed a high-fat diet, providing necessary references for actual production practice.

## 2. Materials and Methods

### 2.1. Experimental Diets

The experimental diets were formulated using fishmeal, *clostridium autoethanogenum* protein (CAP), soybean meal, and low-gossypol cottonseed meal as protein sources and fish oil, soybean oil, and lecithin oil as lipid sources. After all the raw materials were crushed and sifted through a 60-mesh sieve, each ingredient was accurately weighed according to the formula, mixed evenly using a stepwise amplification method, and then each oil source was added. After manually rubbing and sifting, the mixture was placed in a V-type vertical mixer and mixed for 15 min. Then, distilled water (280–350 mL/kg) and choline were added and mixed uniformly. Finally, the mixture was processed into 2.5 mm diameter pellets using a twin-screw extruder (F-26, South China University of Technology), air-dried at room temperature, packed in sealed bags, and stored at −20 °C in a refrigerator for later use. The feed formula and nutritional components are shown in Table 1. Four feeding frequencies were set up, which were 1 time/day, 2 times/day, 3 times/day, and 4 times/day.

### 2.2. Fish and Feeding Trial

The grouper was purchased from the Hongyun Nursery at the southeast dock in Zhanjiang City, Guangdong Province, and then transported to the Marine Biological Research Base of Guangdong Ocean University on Donghai Island, Zhanjiang City. After the grouper was brought back to the base, they were placed in a disinfected 5 m × 4 m × 1.8 m concrete pond for temporary rearing. During this period, they were fed commercial feed twice a day until the fry reached the experimental specifications. At the start of the experiment, after fasting for 24 h, 360 healthy and evenly distributed juvenile fish with an average body mass of (11.51 g ± 0.02) were selected and randomly divided into 4 groups, each with 3 replicates, with each replicate in a 0.3 m^3^ fiberglass bucket, and 30 fish per bucket. During the experiment, feeding was performed manually, and daily feed intake was recorded. The feeding times were set as follows: 1 time/day group, 8:00; 2 times/day group, 8:00, 17:00; 3 times/day group, 8:00, 12:30, 17:00; 4 times/day group, 8:00, 11:00, 14:00, 17:00. During the experiment, aeration was continuously supplied, and water was changed once a day, with a change volume of 70% each time. The water temperature during the experimental period was 28~32 °C, salinity was 28 ppt, dissolved oxygen was >7.1 mg/L, and the ammonia nitrogen content was about 0.05 mg/L.

### 2.3. Sample Collection

At the end of the experiment, all experimental groups were fasted for 24 h before sampling. The grouper in each bucket were weighed and counted to calculate growth performance indicators such as weight gain (WGR) and specific growth rate (SGR). Two fish were removed from each bucket and stored at −20 °C to obtain body composition, crude protein, crude lipid, and moisture. Then, three fish from each bucket were randomly dissected to take their intestines, which were placed in 1 mL explosion-proof tubes for the detection of digestive and antioxidant enzyme activities. Then, the intestines of another four fish from each tank were quickly removed and loaded in 2 mL enzyme-free centrifuge tubes containing RNA later and stored at -80 °C for subsequent analysis of related gene expression. The intestines of another three fish were immediately removed, after being washed by PBS, fixed in 4% formaldehyde over 4 h for histology analysis.

### 2.4. Methods of Analysis

The nutritional components of whole fish and muscle were determined according to the standard methods of the AOAC [16]. Moisture content was measured by drying at 105 °C to a constant weight. Crude protein content was determined using a Skalar Dumas (TN/TC/IC/TOC automatic analyzer, Breda, The Netherlands). Crude fat content was measured by the Soxhlet extraction method (with petroleum ether as the extraction solvent). The crude ash content was determined by incineration in a muffle furnace at 550 °C.

Intestinal superoxide dismutase (SOD), catalase (CAT), total antioxidant capacity (T-AOC), amylase, lipase, and malondialdehyde (MDA), and trypsin were measured using assay kits (from Nanjing Jiancheng Bioengineering Institute, Nanjing, China), strictly following the instructions provided.

Intestinal HE sections were prepared by Wuhan Saiwei Biotechnology Co., Ltd (Wuhan, China). The sections were then observed using a fluorescence inverted microscope (Nikon Eclipse Ti-E, Tokyo, Japan), photographed with LAS3.8 software, and the villus height, villus width, muscle layer thickness, and the number of goblet cells were measured.

### 2.5. Calculation Formula

Weight gain, WGR, % = (M2 − M1)/M1 × 100;

Specific gain rate, SGR, %/d = (lnM2 − lnM1)/t × 100;

Feed conversion ratio, FCR = I/(M4 − M3).

In the formula, M1 and M2 represent the average body mass of the fish at the beginning and end of the experiment (g); t is the experimental period (days); I is the total dry mass of feed consumed (g); M3 and M4 are the total weight of the fish at the beginning and end of the experiment (g).

### 2.6. Intestinal RNA Extraction, cDNA Synthesis, and RT-qPCR

There are three main key steps: RNA extraction, cDNA synthesis, and real-time fluorescence quantitative PCR, which is similar to the specific operation of my last experiment [17]. When extracting RNA in order to avoid degradation, it is necessary to operate on ice and move quickly. RNA was extracted and reverse transcribed using TransZol UP (TransGen Biotech, Beijing, China) and the Evo M-MLV Reverse Transcription Kit (Accurate Biology, Changsha, China). The housekeeping and target genes were designed according to the parental species, and the primers (Table 2) were verified for extension efficiency, and after meeting the requirements, real-time fluorescence quantitative PCR was performed, and the gene expression levels were determined using the 2^−ΔΔCT^ method.

### 2.7. Statistical Analysis

The experimental data were analyzed using one-way ANOVA with SPSS 21.0 software. If significant differences were found, Tukey’s post hoc test was conducted for multiple comparisons. A difference was considered significant at *p* < 0.05, and the experimental data were expressed as “mean ± standard error”.

## 3. Results

### 3.1. Effect of Feeding Frequency on Growth Performance of Hybrid Grouper

**Figure 1:**
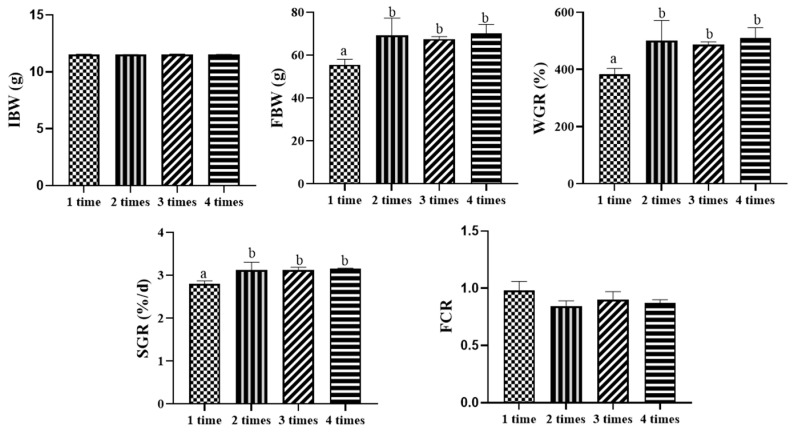
Effect of feeding frequency on growth performance of hybrid grouper. Distinct letters assigned to the bars represent significant differences using Tukey’s test (*p* < 0.05).

The results, as shown in Figure 1, indicate that feeding frequency had a significant impact on final body weight (FBW), specific growth rate (SGR), and weight gain (WGR). With the increase in feeding frequency, there was a trend of FBW, WGR, and SGR to rise and then stabilize, all having the minimum values in the 1 time/day group and significantly lower than the other groups (*p* < 0.05), with no significant differences between the 2 times/day, 3 times/day, and 4 times/day groups (*p* > 0.05). FCR was maximal at 1 time/day, and there was no significant difference between the groups (*p* > 0.05).

### 3.2. Effect of Feeding Frequency on Whole-Body Composition of Hybrid Grouper

The results, as shown in Table 3, indicate that crude protein tended to increase and then stabilize with the increase in feeding frequency. The 1 time/day group was significantly lower than the other groups (*p* < 0.05), while there were no significant differences between the 2 times/day, 3 times/day, and 4 times/day groups (*p* > 0.05). As feeding frequency increases, crude lipid tended to rise, with the 4 times/day group being significantly higher than the other groups (*p* < 0.05), and there were no significant differences between the 1 time/day, 2 times/day, and 3 times/day groups (*p* > 0.05). The increase in feeding frequency led to a decreasing trend in crude ash, with the 1 time/day group being significantly higher than the other groups (*p* < 0.05), and no significant differences between the 2 times/day, 3 times/day, and 4 times/day groups. Moisture content was high in the 1 time/day group and then stabilized (*p* < 0.05).

### 3.3. Effect of Feeding Frequency on Intestinal Digestive Enzyme of Hybrid Grouper

As shown in Table 4, with the increase in feeding frequency, both intestinal amylase (AMS) and lipase (LPS) showed a significant upward trend (*p* < 0.05). AMS had the minimum value in the 1 time/day group, significantly lower than the 3 times/day and 4 times/day groups *(p* < 0.05), and the maximum value in the 4 times/day group, significantly greater than the 2 times/day group (*p* < 0.05), but with no significant difference compared to the 3 times/day group (*p* > 0.05), and no significant difference between the 1 time/day and 2 times/day groups (*p* < 0.05). LPS had the minimum value in the 1 time/day group, significantly lower than the other groups (*p* < 0.05), and the maximum value in the 4 times/day group, significantly greater than the 2 times/day group (*p* < 0.05), but with no significant difference compared to the 3 times/day group and no significant difference between the 2 times/day and 3 times/day groups (*p* > 0.05). Trypsin (TPS) showed a trend of first decreasing and then increasing, with the minimum value in the 2 times/day group, significantly lower than the other groups (*p* < 0.05), and the maximum value in the 4 times/day group, significantly greater than the 1 time/day group (*p* < 0.05), with no significant difference compared to the 3 times/day group (*p* > 0.05), and no significant difference between the 1 time/day group and the 3 times/day group *(p* < 0.05).

### 3.4. Effect of Feeding Frequency on Intestinal Antioxidant Enzyme Activity of Hybrid Grouper

As indicated in Table 5, superoxide dismutase (SOD) was low in the 1 time/day group and 3 times/day and significantly lower than the 2 times/day and 4 times/day groups (*p* < 0.05). Both T-AOC and glutathione peroxidase (GPX) had their minimum values in the 1 time/day group, significantly lower than the other groups (*p* < 0.05), with no significant differences among the 2 times/day, 3 times/day, and 4 times/day groups (*p* > 0.05). Catalase (CAT) had its maximum value in the 4 times/day group, significantly higher than the other groups (*p* < 0.05), with no significant differences among the 1 time/day, 2 times/day, and 3 times/day groups.

### 3.5. Effect of Feeding Frequency on Intestinal Structure of Hybrid Grouper

**Figure 2:**
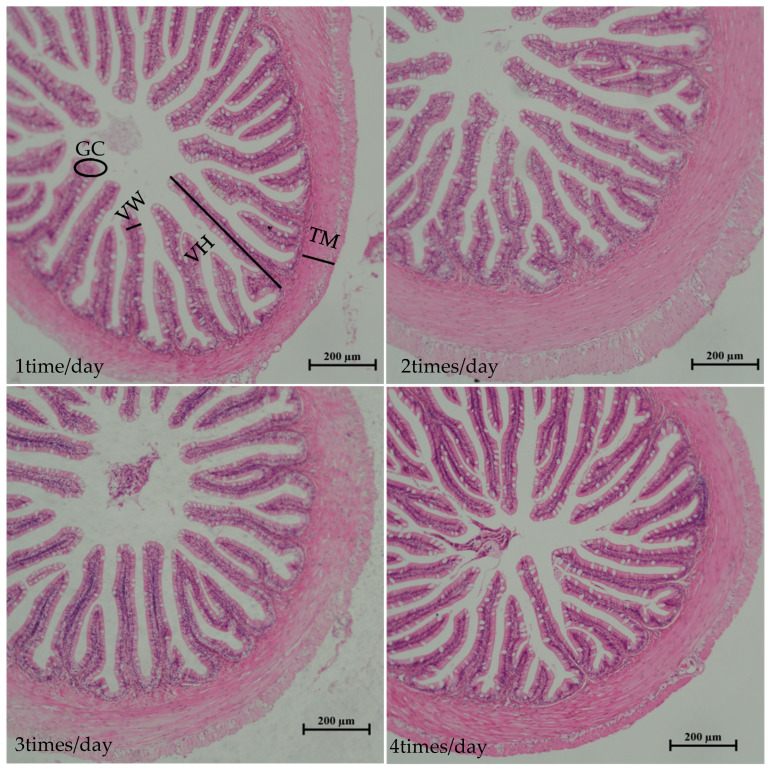
Effect of feeding frequency on intestinal structure of hybrid grouper.

As the feeding frequency increases, the villus height (VH), muscular thickness (MT), and goblet cell (GC) showed a trend of first increasing and then decreasing (Figure 2), with the 2 times/day group having the maximum values, significantly greater than the other groups (*p* < 0.05). VH showed no significant difference among the 1 time/day, 3 times/day, and 4 times/day groups (*p* > 0.05), while MT was significantly greater in the 3 times/day group compared to the 1 time/day and 4 times/day groups (*p* < 0.05), with no significant difference between the 1 time/day and 4 times/day groups (*p* > 0.05). For GC, the 1 time/day group was significantly greater than the 4 times/day group (*p* < 0.05) but showed no significant difference compared to the 3 times/day group (*p* > 0.05), and there was also no significant difference between the 3 times/day and 4 times/day groups (*p* > 0.05). The villus width (VW) showed a decreasing trend, with the 4 times/day group having the minimum value, significantly less than the 1 time/day and 2 times/day groups (*p* < 0.05), and no significant difference among the 1 time/day, 2 times/day, and 3 times/day groups (*p* > 0.05), (see Table 6).

### 3.6. Effect of Feeding Frequency on Intestinal Antioxidant and Inflammation-Related Genes of Hybrid Grouper

**Figure 3:**
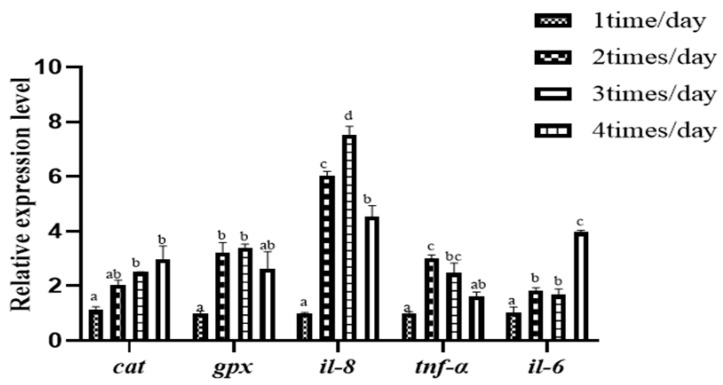
Effect of feeding frequency on intestinal antioxidant and immune-related genes of hybrid grouper. Distinct letters assigned to the bars represent significant differences using Tukey’s test (*p* < 0.05).

As shown in Figure 3, with the increase in feeding frequency, the expression levels of *cat* and *il-6* showed an upward trend, with the 1 time/day group significantly lower than the other treatment groups (*p* < 0.05). *il-8* and *tnf-α* exhibit a trend of first increasing and then decreasing; the 1 time/day group is significantly lower than the other treatment groups (*p* < 0.05). *gpx* also showed a trend of first increasing and then decreasing, but the decrease was not significant *(p* > 0.05). The 1 time/day group was significantly smaller than the 2 times/day and 3 times/day groups (*p* < 0.05).

## 4. Discussion

The experimental results indicate that feeding frequency has a significant impact on the growth performance of the hybrid grouper. As the feeding frequency increases, there is a trend of FBW, WGR, and SGR to rise and then stabilize, with the 1 time/day group showing the lowest values. This suggests that appropriately increasing the feeding frequency can promote the growth of the grouper. The reason might be that an increase in feeding frequency affects the feeding rate, leading to better absorption and digestion of food by the fish [18]. This outcome is similar to the findings from studies on hybrid bream (*Megalobrama terminalis Richardson* ♀ × *Erythroculter ilishaeformis* ♂) [14] and catfish (*Silurus meridionalis*) [19]. However, when the feeding frequency exceeds twice a day, there is no significant increase in FBW, WGR, and SGR, but there is a trend of FCR (feed conversion ratio) to rise. This indicates that a higher feeding frequency is not always better; exceeding the optimal feeding frequency does not yield higher FBW, WGR, and SGR but instead increases FCR, thereby reducing the benefits of aquaculture. Similar results have been observed in studies on large yellow croaker (*Pseudosciaena crocea*) [15], grass carp [20], and hybrid sturgeon (*Acipenser schrenckii Brandt*♀ × *A. baeri Brandt*♂) [21]. The reason might be that an increased feeding frequency leads to shorter intervals between meals at 3 times/day and 4 times/day, causing the feed in the stomach and intestines not to be completely emptied before new food is ingested again. This can lead to a reflexive rapid movement of intestinal contents towards the posterior part of the intestine, resulting in the expulsion of undigested feed, which in turn affects the digestibility of nutrients in the feed [22].

The whole-body chemical composition is commonly used as a standard for assessing the nutritional quality of fish [23]. The results of this experiment indicate that feeding frequency significantly affects the whole-body chemical composition. As feeding frequency increases, crude protein tends to rise and then stabilize, while crude lipid shows an increasing trend, with the highest value at 4 times/day. Ash tends to decrease and then stabilize, with the highest value in the 1 time/day group. These results are similar to those found in studies on large yellow croaker (*Pseudosciaena crocea*) [18], *Schizothorax wangchiachii* [24], and *Clarias gariepinus* [19]. The explanation for this outcome in the study of large yellow croaker is that fish fed at a lower frequency expend more energy to compete for limited food or to prevent cannibalism, both of which can accelerate protein and lipid metabolism, leading to a reduction in body protein and lipid content. In contrast, a higher feeding frequency enhances the accumulation of protein and lipid in fish [18]. The moisture content shows a trend of decreasing and then stabilizing with an increase in feeding frequency, with the highest value in the 1 time/day group, a result that is similar to the findings in the study of *Pseudobagrus ussuriensis* [25].

The activity of intestinal digestive enzymes can be used to assess the ability of fish to digest and absorb nutrients [26,27]. TPS is one of the most important digestive enzymes in aquatic animals, primarily responsible for the hydrolysis of most proteins in feed. LPS mainly acts on most of the fats in feed, and AMS is a type of carbohydrate-hydrolyzing enzyme and an important digestive enzyme in aquatic animals. The results of this experiment show that feeding frequency has a significant impact on digestive enzymes. The activity of TPS in the 1 time/day group was significantly higher than in the 2 times/day group. The reason may be that the 1 time/day group has a longer interval between meals, requiring increased TPS activity to fully digest and absorb nutrients [23]. This result is similar to the findings in studies on Russian sturgeon (*Acipenser gueldenstaedti*) [21] and Vachell’s yellow catfish (*Pelteobagrus vachelli*) [28]. The digestive enzyme activities in the 3 times/day and 4 times/day groups are greater than in the 2 times/day group. The reason may be that the 3 times/day and 4 times/day groups have shorter intervals between meals, and the feeding rate after each meal is greater than in the 2 times/day group, thus necessitating a higher digestive enzyme activity to cope with the increased intake.

TAOC is a comprehensive index reflecting the overall antioxidant capacity of the enzymatic and non-enzymatic antioxidant systems of the body. T-AOC not only includes the activities of antioxidant enzymes such as SOD, CAT, and glutathione peroxidase (GPX) but also includes the levels of non-enzymatic antioxidants such as vitamin C, vitamin E, and glutathione (GSH). A higher T-AOC value indicates a stronger antioxidant capacity of the animal body and a stronger ability to scavenge free radicals. CAT is an important antioxidant enzyme that can decompose hydrogen peroxide (H_2_O_2_) into water and oxygen, thereby reducing the toxic effect of hydrogen peroxide on cells. CAT is widely present in cells, especially in hepatocytes and erythrocytes at high levels. The high expression of CAT means that the body is able to remove hydrogen peroxide more effectively and reduce cell damage caused by oxidative stress. SOD is an antioxidant enzyme that can specifically scavenge superoxide anion (O_2_^−^). SOD reduces the toxicity of superoxide anion by dismutating it to hydrogen peroxide and oxygen. The expression level of SOD in cells is closely related to the antioxidant capacity of the body, and the high expression of SOD can better protect cells from oxidative stress. GPX is a glutathione (GSH)-dependent antioxidant enzyme that reduces hydrogen peroxide and lipid peroxides to water and corresponding alcohols, thereby protecting cell membranes and organelles from oxidative damage. The expression level of GPX in cells is positively correlated with the antioxidant capacity of the body. High expression of GPX can more effectively remove peroxides and reduce oxidative stress damage to cells. They clear free radicals in the body through different mechanisms to maintain the REDOX balance of cells [29]. The integrity of intestinal structure plays an important role in maintaining the normal digestive and absorptive capabilities of the intestine, as well as its barrier function [30]. Histological sections of the intestine (HE staining) can be used to assess intestinal health [31], where VH and VW are related to the intestinal absorption area [32], and TM is related to the intestinal motility, reflecting the motility capacity of the intestine [33]. CG are highly polarized columnar epithelial cells widely distributed in the digestive tract, and their number can reflect the quality of mucus [34].The findings of this experiment indicate that the T-AOC, CAT, SOD, and GPX levels in the 1 time/day group were relatively low, and the VH and TM were also low, suggesting that the intestines in the 1 time/day group were under oxidative stress and that the intestinal structure was damaged. This could be the reason why the digestive enzyme levels were lower in the 1 time/day group. In contrast, the 2 times/day group showed higher levels of VH, VW, TM, CG, T-AOC, CAT, SOD, and GPX, indicating intact intestinal structure, better antioxidant capacity, and improved digestive and absorptive capabilities. However, when the feeding frequency exceeded 2 times/day, there was a significant downward trend in VH, TM, and CG, suggesting that exceeding the optimal feeding frequency could pose a risk of intestinal structural damage. When the feeding frequency is too low, the feed intake and intestinal digestion ability of groupers are too weak, and the intestinal burden of groupers is also increased when the feeding frequency is too high.

*il-8* belongs to the CXC chemokine family and is a small molecular inflammatory cytokine. During inflammation or infection, *il-8* is mainly produced by macrophages, epithelial cells, endothelial cells, and various other cell types as a response to these pathological conditions [35]. *il-6* has a broad range of biological activities and is involved in many physiological and pathological processes, including immune regulation, inflammatory responses, cell growth, and the onset of diseases [36]. *tnf-α* is one of the key pro-inflammatory cytokines that can activate inflammatory cells [37]. The *cat* gene encodes catalase, a key component of the cellular antioxidant defense system, which prevents oxidative stress and cell damage by breaking down hydrogen peroxide (H_2_O_2_) [38]. GPX is essential for the antioxidant defense system, neutralizing H_2_O_2_ and other harmful organic hydroperoxides by using glutathione (GSH) as an electron donor, thereby preventing oxidative damage to cells. The experiment found that as the feeding frequency increases, the relative expression of intestinal *cat* also increases, indicating that the intestinal antioxidant capacity is enhanced with an increased feeding frequency. When the feeding frequency is greater than once a day, the relative expression of pro-inflammatory genes such as *il-6*, *il-8*, and *tnf-α* is significantly upregulated. If the expression levels are too high, it may trigger an inflammatory response [39]. Therefore, this suggests that an increase in feeding frequency may also carry the risk of inducing intestinal inflammatory reactions.

## 5. Conclusions

In conclusion, a 1 time/day feeding frequency of a high-fat diet reduced growth performance and health of hybrid grouper. Based on the criteria of growth performance, enzyme activity, and morphology, it is recommended that the high-fat diet be fed 2 times/day in practice.

## Figures and Tables

**Table 1:** Composition and proportion of basic diets (dry matter%).

Items	Content
Fish meal	35
Soybean meal	5
*Clostridium autoethanogenum* protein	10.6
Low-gossypol cottonseed meal	10
Wheat flour	18
Soybean lecithin	1.5
Fish oil	6.6
Soybean oil	3.3
Ca(H_2_PO_4_)_2_	1.5
Choline chloride	0.5
Compound premix ^a^	1
Vitamin C	0.05
Met	0.5
Arg	0.3
Microcrystalline cellulose	5.95
Antioxidant	0.05
Attractant	0.15
Total	100
Proximate composition ^b^	
Moisture	7.9
Crude protein	44.1
Crude lipid	15.4

^a^ Compound premix was obtained from Qingdao Master Biotechnology Co, Ltd (Qingdao, China). ^b^ Measured value.

**Table 2:** Real-time quantitative PCR primers.

Gene	Primer Sequence (5′ to 3′)	GenBank Accession No.
*β-actin*-F/R	ACTGCTGCCTCCTCTTCATC/	KU746361.1
	ACCGCAAGACTCCATACCAA	
*il-6*-F/R	F: AGGAAGTCTGGCTGTCAGGA R: GCCCTGAGGCCTTCAAGATT	JN806222.1
*il-8*-F/R	F: AAGTTTGCCTTGACCCGAA R: AAGCAGATCTCTCCCGGTCT	GU988706.1
*tnf-α*-F/R	F: GTGGCCTACACGACTGCACC R: TACAAAGGGCCACAGTGAGA	FJ491411.1
*gpx*-F/R	F: TCCTCTGTGGAAGTGGCTGA R: TCATCCAGGGGTCCGTATCT	HQ441085.1
*cat*-F/R	F: ACCTATTGCTGTCCGCTTCTC R: GTGGATGAAGGACGGGAACA	AY735009.1

Notes: *il-6*, interleukin-6; *il-8*, interleukin-8; *tnf-α*, tumor necrosis factor-alpha; *gpx*, glutathione peroxidase; *cat*, catalase.

**Table 3:** Effect of feeding frequency on whole-body chemical composition of hybrid grouper.

Items	Groups (Dry Matter %)			
	1 Time	2 Times	3 Times	4 Times
Crude protein	56.57 ± 0.09 ^a^	57.33 ± 0.20 ^b^	57.22 ± 0.04 ^b^	57.38 ± 0.05 ^b^
Crude lipid	25.36 ± 0.12 ^a^	26.26 ± 0.04 ^a^	26.24 ± 0.21 ^a^	27.45 ± 0.59 ^b^
Crude ash	17.35 ± 0.26 ^b^	16.39 ± 0.17 ^a^	16.66 ± 0.20 ^a^	16.73 ± 0.34 ^a^
Moisture	72.38 ± 0.42 ^b^	71.31 ± 0.25 ^a^	71.09 ± 0.08 ^a^	71.23 ± 0.12 ^a^

Note: The data in the table are represented by mean ± standard error (n = 3). No letters or identical letters in the superscript on the same line indicate no significant differences (*p* > 0.05). Different letters indicate significant differences (*p* < 0.05).

**Table 4:** Effect of feeding frequency on intestinal digestive enzymes of hybrid grouper.

Items	Groups			
	1 Time	2 Times	3 Times	4 Times
Trypsin (U/mg prot)	1960.45 ± 117.47 ^b^	1517.31 ± 37.28 ^a^	2102.25 ± 63.81 ^bc^	2269.73 ± 29.06 ^c^
Lipase (mU/mg prot)	520.41 ± 39.69 ^a^	726.92 ± 35.83 ^b^	911.09 ± 99.65 ^bc^	983.82 ± 38.67 ^c^
Amylase (mU/mg prot)	320.76 ± 12.00 ^a^	358.22 ± 5.74 ^ab^	394.12 ± 20.54 ^bc^	438.17 ± 16.73 ^c^

Note: The data in the table are represented by mean ± standard error (n = 3). No letters or identical letters in the superscript on the same line indicate no significant differences (*p* > 0.05). Different letters indicate significant differences (*p* < 0.05).

**Table 5:** Effect of feeding frequency on intestinal antioxidant enzyme activity of hybrid grouper.

Items	Groups			
	1 Time	2 Times	3 Times	4 Times
SOD (ng/mg prot)	20.66 ± 1.22 ^a^	26.20 ± 0.90 ^b^	22.96 ± 1.68 ^a^	27.55 ± 0.64 ^b^
T-AOC (umol trolox/mgProt)	0.34 ± 0.01 ^a^	0.41 ± 0.01 ^b^	0.41 ± 0.03 ^b^	0.47 ± 0.04 ^b^
CAT (ng/mg prot)	13.24 ± 0.92 ^a^	16.02 ± 0.39 ^a^	15.57 ± 0.95 ^a^	20.51 ± 1.41 ^b^
GPX (ng/mg prot)	68.99 ± 0.92 ^a^	113.20 ± 6.50 ^b^	122.27 ± 12.33 ^b^	111.02 ± 7.27 ^b^
MDA (ng/mg prot)	11.89 ± 2.72	11.99 ± 2.97	12.12 ± 2.19	12.28 ± 2.40

Note: The data in the table are represented by mean ± standard error (n = 3). No letters or identical letters in the superscript on the same line indicate no significant differences (*p* > 0.05). Different letters indicate significant differences (*p* < 0.05).

**Table 6:** Effect of feeding frequency on intestinal structure of hybrid grouper.

Items	Groups			
	1 Time	2 Times	3 Times	4 Times
VH (μm)	355.38 ± 2.00 ^a^	506.56 ± 13.26 ^b^	382.05 ± 8.15 ^a^	388.14 ± 17.56 ^a^
VW (μm)	61.60 ± 0.69 ^b^	61.89 ± 2.07 ^b^	58.20 ± 0.77 ^ab^	54.13 ± 2.16 ^a^
MT (μm)	105.99 ± 2.14 ^a^	185.42 ± 1.95 ^c^	149.27 ± 14.62 ^b^	120.50 ± 4.70 ^a^
GC (number)	19.25 ± 1.31 ^b^	24.25 ± 0.47 ^c^	16.75 ± 0.62 ^ab^	15.00 ± 0.41 ^a^

Abbreviations: muscular thickness (MT); villus height (VH); villus width (VW); goblet cell (GC). Note: The data in the table are represented by mean ± standard error (n = 3). No letters or identical letters in the superscript on the same line indicate no significant differences (*p* > 0.05). Different letters indicate significant differences (*p* < 0.05).

## Data Availability

The data that support the findings of this study are available on request from the corresponding author. The data are not publicly available due to privacy or ethical restrictions.
